# Prognostic value and molecular mechanism of photodynamic therapy and apoptosis related gene FGFR1 in bladder cancer

**DOI:** 10.3389/fonc.2025.1578695

**Published:** 2025-09-10

**Authors:** Lei Wang, Yanchun Wang, Yanqing Wang, Zhengwei Wang, Guangzhou Cheng, Zhunan Xu, Xiaoqiang Liu

**Affiliations:** ^1^ Department of Urology, Tianjin Medical University General Hospital, Tianjin, China; ^2^ Department of Urology, Tengzhou Central People’s Hospital, Tengzhou, Shandong, China

**Keywords:** bladder cancer (blca), photodynamic therapy (PDT), apoptosis, FGFR1, prognostic

## Abstract

**Purpose:**

Photodynamic therapy (PDT) is an innovative non-invasive therapy for human cancer treatment. The significance of apoptosis-related genes (ARGs) in the prognosis of bladder cancer (BLCA) has gradually emerged. Therefore, this study aims to investigate the prognostic significance and pathogenesis of PDT related genes (PDTRGs)-ARGs in BLCA cases.

**Methods:**

Based on the BLCA data in TCGA, PDTRGs-ARGs with prognostic value in BLCA patients were screened. Subsequently, the prognostic value and diagnostic performance of all candidate genes were evaluated by univariate Cox regression analysis and ROC curves. Then, GSEA, GSVA and immune microenvironment analysis were conducted based on candidate genes. Finally, the molecular mechanisms of key candidate genes in BLCA patients were initially explored by qRT-PCR, CCK-8 analysis, Transwell Assay and Western Blotting.

**Results:**

A total of 5 ARGs-PDTRGs (EMP1, FGFR1, PLPPR4, JUN, TNFRSF25) were screened as prognostic biomarkers for BLCA. Survival analysis revealed significant differences in overall survival of the five prognostic biomarkers in the high/low expression groups. ROC curve analysis revealed that the five prognostic biomarkers had strong prognostic predictive ability. QRT-PCR proved that the expression of EMP1, FGFR1, PLPPR4 and JUN was obviously reduced, while TNFRSF25 was markedly increased in BLCA tissue samples and cell lines. The following research confirmed that FGFR1 inhibited the biological process of T24 cells by activating cGMP-PKG pathway.

**Conclusion:**

Five ARGs-PDTRGs (EMP1, FGFR1, PLPPR4, JUN, TNFRSF25) were screened as prognostic biomarkers for BLCA. Among them, FGFR1 inhibits the biological process of T24 cells via activating cGMP-PKG pathway.

## 1 Introduction

Bladder cancer (BLCA) has a high incidence and mortality, which brings a huge economic burden to the patient’s family and society (1). Smoking, occupational exposure and schistosomiasis infection are the main risk factors for BLCA (2). Studies have shown that BLCA accounts for 5%-10% of all cancers in men, with a higher incidence than in women (3). BLCA mainly occurs in the urothelial epithelium, including myogenic bladder cancer (MIBC) and non-myogenic prostate cancer (NMIBC). Among them, NMIBC accounts for about 4/5 of BLCA, and its treatment strategy is mainly radical treatment combined with systemic therapy (4, 5). However, detection of early-stage tumors is still insufficient, and current treatment strategies have not significantly improved the overall survival rate of BLCA patients (6). Accordingly, it is necessary to explore novel prognostic biomarkers in BLCA tumorigenesis, so as to provide new treatment options for BLCA patients (7).

Photodynamic therapy (PDT) is a non-invasive and innovative therapy that utilizes photosensitizers to generate reactive oxygen species clusters upon photoactivation, thereby inducing targeted cell death (8, 9). PDT is well tolerated in clinical patients and is increasingly used in the treatment of human cancers, including bladder cancer (10, 11). For example, the photosensitizer 5-aminolevulinic acid (5-ALA) has been studied for screening NMIBC patients before and after transurethral resection of bladder tumors (TURBT) (12). Currently, PDT is considered an important treatment option for combination therapy (13). PDT was combined with intravesical chemotherapy or systemic therapy to improve local tumor control (11). Therefore, further research and exploration of PDT will contribute to the development of new treatment plans for BLCA.

Apoptosis pathway is a basic biological phenomenon that occurs regularly in cells and plays a key role in maintaining cell and tissue homeostasis (14, 15). However, if this balance is disrupted, it will directly or indirectly lead to the occurrence of a variety of diseases (16). More and more studies are devoted to exploring the value of apoptosis-related genes (ARGs) in cancer (17, 18). One study found that YWHAQ, MAP2K1, EGFR and SCAPN14 were related to the prognosis of BLCA (19). Although scholars are paying increasing attention to the function of ARGs in various human tumors, the prognostic value of PDT associated genes (PDTGs) -ARGs in BLCA remains unclear. Therefore, this research aims to investigate the prognostic significance and its mechanism of PDTGs-ARGs in BLCA patients.

For this study, differentially expressed ARGs-photodynamic therapy related genes (PDTRGs) in BLCA patients were screened from numerous cohorts, and genes with prognostic value were further identified and validated by least absolute shrinkage and selection operator (LASSO) regression analysis. Besides, the mechanism and immune microenvironment of prognostic markers were explored. Moreover, the expression level of prognostic markers was validated in BLCA tissue samples and cell lines. It is worth noting that we also preliminarily explored the pathway of FGFR1 in BLCA. This research aimed to identify novel prognostic biomarkers, explore their role in the pathogenesis of BLCA, understand their relationship with the immune microenvironment, and lay the foundation for prolonging the survival of BLCA patients.

## 2 Methods

### 2.1 Differential expression genes identification

The mRNA expression profile, clinical information and survival information of BLCA were obtained from The Cancer Genome Atlas (TCGA) database as a training set. After pretreatment, 398 BLCA specimens and 19 normal control specimens were retained. The GSE13507 dataset was obtained from the Gene Expression Omnibus (GEO) database as a validation set, including 188 BLCA specimens and 68 normal specimens. Besides, the genes related to photodynamic therapy were searched in GeneCards database with the keyword “Photodynamic therapy”, and screened using a relevance score>0.7 as the threshold, resulting in a total of 210 PDTRGs. Moreover, a search was conducted in the MSigDB database using the keyword “Apoptosis”, and five sets of ARGs were downloaded, including “HALLMARK-APOPTOSIS.v2024.1.Hs”, “ALCALA-APOPTOSIC.v2024.1.Hs”, “KEGG-APOPTOSIS.v2024.1.Hs”, “WP-APOPTOSIS.v2024.1.Hs”, and “REACTOME-APOPTOSIS.v2024.1.Hs”, and a total of 463 ARGs were obtained after deduplication.

Next, R package “limma” (20, 21)was used to analyze the DEGs between tumor specimens and normal specimens in the TCGA-BLCA datase.

### 2.2 Weighted gene co-expression network analysis and candidate gene screening

The samples were grouped as traits and analyzed by R package “WGCNA” to screen BLCA-related module genes. The module most related to BLCA was selected as the key module, screen the crucial genes in the key module according to the criteria of |GS|>0.2, and recorded as the BLCA-related module genes. Next, DEGs intersect with ARGs and PDTRGs respectively to obtain DEGs-ARGs and DEGs-PDTRGs. Then the pearson correlation (cor) between the two genes was calculated, and all DEGs-ARGs and DEGs-PDTRGs with |cor| > 0.3 were denoted as DEGs-ARGs-PDTRGs. Then, take the intersection of DEGs-ARGs-PDTRGs and the module genes obtained from WGCNA analysis, and record them as candidate genes for subsequent analysis.

### 2.3 Functional enrichment analysis and protein-protein interaction (PPI) network analysis

To reveal the potential mechanisms of candidate genes, Gene Ontology (GO) and Kyoto Encyclopedia of Genes and Genomes (KEGG) functional enrichment of the above candidate genes were conducted using the R-package “clusterProfiler” (22). In addition, PPI relationship among key genes was obtained through the STRING database, and a PPI network was mapped.

### 2.4 Screening and validation of prognostic biomarkers

To assess the association between key genes and patient survival, univariate Cox regression analysis was conducted on key genes in TCGA-BLCA dataset, and key genes with prognostic value were obtained (*P* ≤ 0.05). The selected genes were then analyzed by LASSO regression and 10x cross-validation was employed to identify prognostic biomarkers in patients (23, 24). Besides, Kaplan-Meier (K-M) survival analysis was employed on the high and low level groups of biomarkers using R package survival. To evaluate the diagnostic performance of biomarkers, receiver operating characteristic (ROC) curves were plotted for the biomarker (25). The expression and diagnosis of the biomarkers were then verified separately in the validation set GSE13507.

### 2.5 GSEA and GSVA enrichment analysis

Based on the MSigDB database, single gene GSEA analysis was conducted on markers in the TCGA-BLCA dataset to investigate the significant enrichment pathway of the biomarker. Besides, 17 immune response pathways in the Immport database were used as background gene sets for enrichment score calculation. R-packet limma difference analysis was used to screen out the different immune response pathways between the two groups of samples, calculate the association between markers and different immune response pathways.

### 2.6 Immune microenvironment analysis

In the training set, CIBERSORT was employed to assess the proportion of immunoinfiltrating cell types in patients. The differences between two groups of immune infiltrating cells were analyzed using Wilcoxon test, and Spearman was applied to analyze the association between biomarkers and immune cells.

### 2.7 Clinical samples and cells

Fifty-four patients who underwent radical resection of BLCA in our hospital were enrolled. During the operation, BLCA tissue specimens and adjacent tissue specimens (≥5 cm from cancer tissue) of all patients were collected. The study was approved by the Clinical Research Ethics Committee of our hospital, and subjects signed informed consent. Human BLCA cell lines T24, J82 and normal uroepithelial cells SV-HUC-1 were obtained from the ATCC Repository in the United States. All cells were incubated in DMEM medium containing double antibody (1×10^5^U/L penicillin, 100mg/L streptomycin) and 10% fetal bovine serum, and then cultured in a 5% CO_2_ incubator at 37 °C. FGFR1 overexpression vector PCDNA3.1-FGFR1 (OE-FGFR1) and negative control pcDNA3.1 vector(NC) were provided via Hanbio (Shanghai, China). Cell transfection was conducted according to the Lipofectamine TM2000 manual, and the cells were incubated for 48 hours for further study.

### 2.8 qRT-PCR

Based on the operation manual, total RNA was isolated from cells and tissues using Trizol reagents. 2μg total RNA was synthesized into cDNA according to the instructions of Prime ScriptTM RT kit, which was employed as a template for qRT-PCR reaction using SYBR Green PCR kit. The relative gene expression was calculated by The 2^-△△Ct^ method was applied to calculate the relative gene expression, and GAPDH as the internal reference.

### 2.9 Cell proliferation analysis

The transfected cell suspension was laid on 96-well plates with a density of 2 × 10^3^ cells each well, and three accessory wells were set up in each group. After cell attachment, marked as 0h, the cells were incubated for 0, 24, 48 and 72 h, respectively. Then, the cells were treated with 10μL CCK-8 reagent and incubated for 2 hours. Measure the absorbance at 450 nm using a microplate reader (TECAN, Mechelen, Belgium).

### 2.10 Transwell assay

Cells were collected from each group, and then and then 500 μ L of cell suspension was joined in the upper chamber of transwell, and 500μL of medium (containing 10% fetal bovine serum) was joined in the lower chamber. The cells were incubated for 24 h at constant temperature, then the cells were fixed with 4% paraformaldehyde for 15 min, stained with crystal violet for 20 min, and the cells in the bright field of vision were observed under an inverted microscope. Invasion assay: Matrigel was diluted in proportion, and 50μL dilution was spread on the bottom of the upper chamber of the transwell and cultured for 5 hours until solidification. The following experiments were performed as migration assay.

### 2.11 Western blotting

Cells in every group were obtained and total protein was extracted via RIPA protein lysate (ProMab Biotechnology, USA). The BCA kit (CWBio) was applied to measure protein concentration, the protein samples were treated via 10% SDS-PAGE gel electrophoresis, and next, move onto PVDF membrane (CWBio) via semi-dry method. After 2 hours of closed culture with 5% skim milk powder, the corresponding diluted primary antibody was joined and cultured at 4°C for 12 h, followed by the diluted secondary antibody was joined and cultured at 37 °C for 2 h. After 3 times of washing with PBS, the chemiluminescent developer ECL was added for development. Using GAPDH as internal reference gene.

### 2.12 Statistical analysis

R 4.3.3, GraphPad Prism 9.5 and SPSS 22.0 were employed for data handling, plotting and statistical analysis. Wilcoxon rank sum test or T test were employed to compare continuous variables. The comparison of categorical variables was performed by chi-square test. Pearson correlation coefficient was applied to assess the correlation between two continuous variables. The R package “pROC” plots the ROC curve and calculates the AUC separately to evaluate the diagnostic performance of each biomarker. Data were shown as mean ± standard deviation (SD). Each group of experiments was repeated three times., and *P* less than 0.05 was supposed obviously significant.

## 3 Results

### 3.1 Screening of DEGs

**Figure 1:**
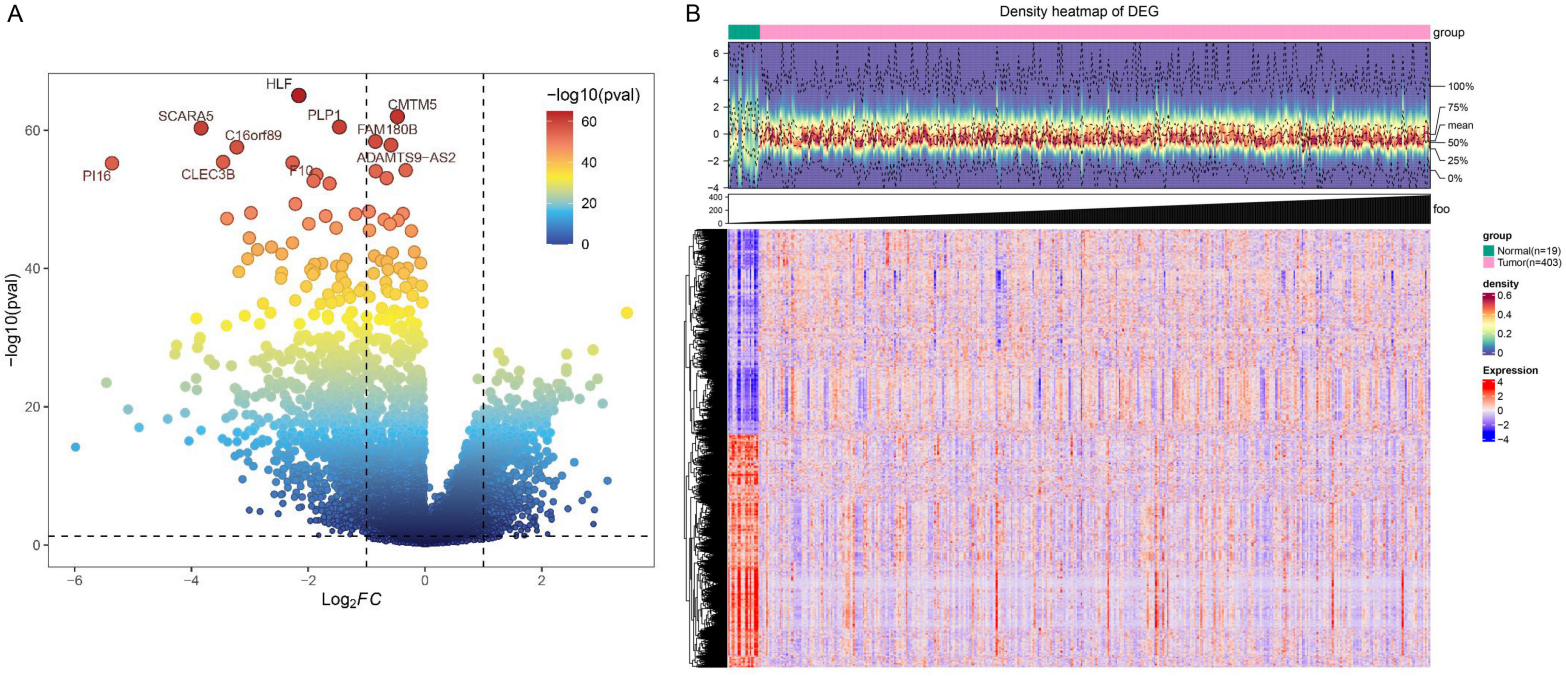
Identification of DEGs. **(A)** Volcanic plot of DEGs. Each dot represents a gene, and the color of the dot represents the significance of the differential gene, ranging from blue to red, and the reder the color, the more significant the gene is. According to the reference line, the genes in the upper right corner were up-regulated DEGs, the genes in the upper left corner were down-regulated DEGs, and the remaining genes were no difference genes. The labeled genes in the figure are the ten most significant genes. **(B)** Heat map of DEGs. In the annotation bar above, green represents normal samples and pink represents tumor samples; the color gradient in the central density heatmap indicates sample expression density, with darker red signifying higher density; the gray bar annotation indicates sample size; in the heatmap below, the x-axis represents samples, the y-axis represents genes, red denotes highly expressed genes, and blue denotes lowly expressed genes.

In this project, a total of 1630 DEGs were screened based on tumor samples and normal samples from the TCGA-BLCA dataset, containing 866 down-regulated genes and 764 up-regulated genes (**Figure 1A**). The heatmap shows the expression of DEGs in tumor specimens and normal specimens, and it can be seen that all DEGs can be well separated by group (**Figure 1B**).

### 3.2 Screening of candidate genes

**Figure 2:**
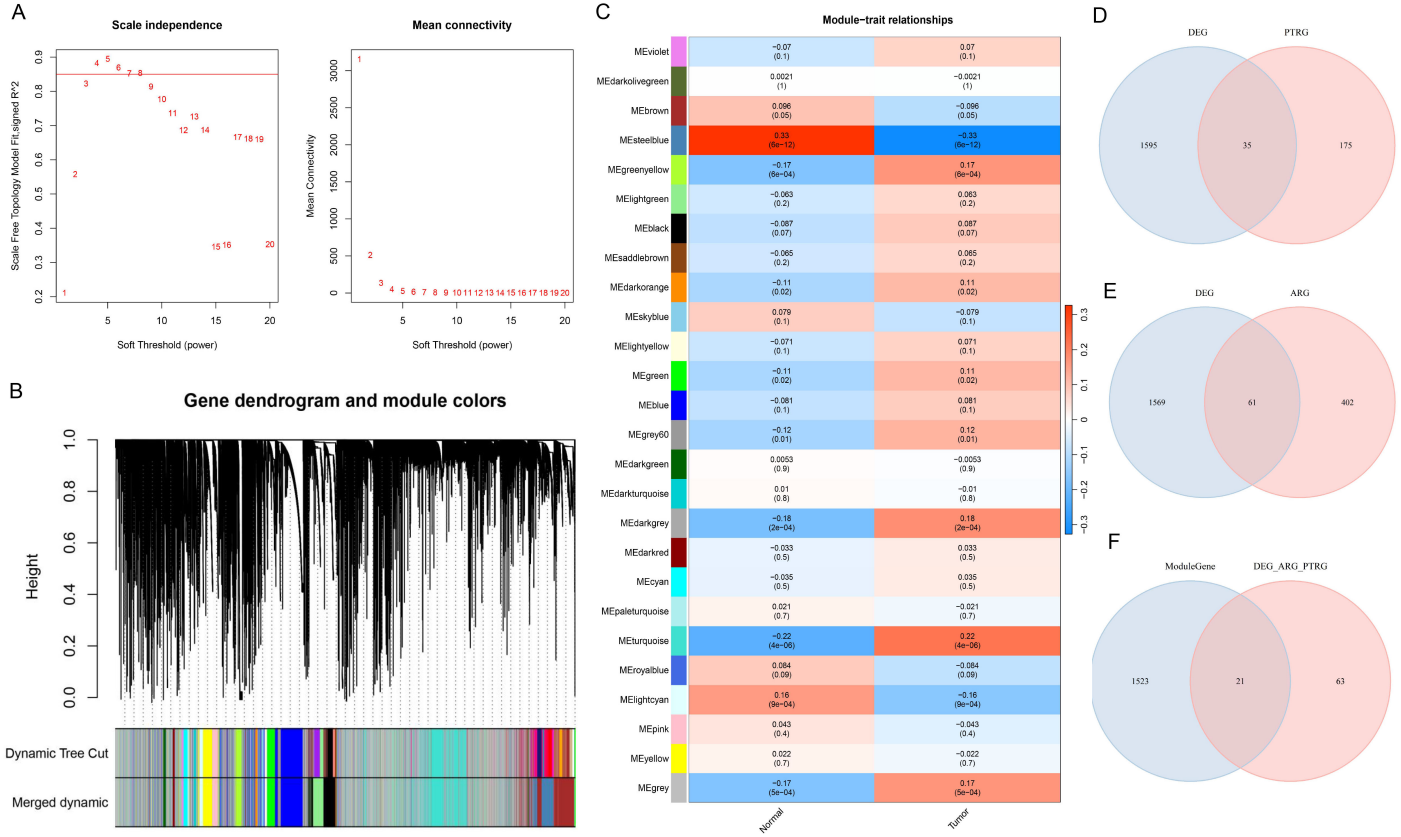
Screening of candidate genes. **(A)** Map of the scale-free soft threshold distribution. When R² reaches 0.85 (marked by the red line), the soft threshold tends to stabilize. At this point, the value 7 is closest to the 0.85 threshold. Additionally, when the mean connectivity approaches 0, the corresponding soft threshold is also 7. Therefore, we selected 7 as the optimal soft threshold. **(B)** Merging graph of gene clustering and module partitioning. Different colors represent different modules, and gray is the default for genes that cannot be classified in any module. **(C)** Heatmap of module and trait correlation. Red represents positive correlation, green represents negative correlation, and the depth of color represents the degree of correlation. **(D)** Venn plot of DEGs and PDTRGs. **(E)** Venn diagram of DEGs and ARGs. **(F)** Venn plot of Model Genes versus DEGs-ARGs-PDTRGs.

In this study, sample grouping was used for WGCNA analysis, and BLCA-related module genes were screened. First, we determined the soft threshold of the data. After calculation, the optimal soft threshold was determined to be 7 (**Figure 2A**). Then, according to the standard of hybrid dynamic shear tree, the modules are divided into 26 modules (**Figure 2B**). As shown in **Figure 2C**, in the correlation heat map, MEsteelblue showed a high correlation with the Tumor group samples (cor=-0.33& *P=*6e-12), and could be used as a key module. 1544 module genes were screened using |GS| > 0.2 as the criterion. To screen for ARGs and PDTRGs significantly associated with BLCA, DEGs were intersected with ARGs and PDTRGs, respectively, resulting in 35 DEGs-ARGs (**Figure 2D**) and 61 DEGs-PDTRGs (**Figure 2E**). Then, pearson correlation between DEGs-ARGs and DEGs-PDTRGs was calculated. Among them, all the DEGs-ARGs and DEGs-PDTRGs with |cor| > 0.3 were selected as DEGS-ARGS-PDTRGs, and there were 84 DEGS-ARGS-PDTRGs. They were intersected with Model Genes, and the 21 intersection genes obtained were used in the next study (**Figure 2F**).

### 3.3 KEGG and GO enrichment analysis

**Figure 3:**
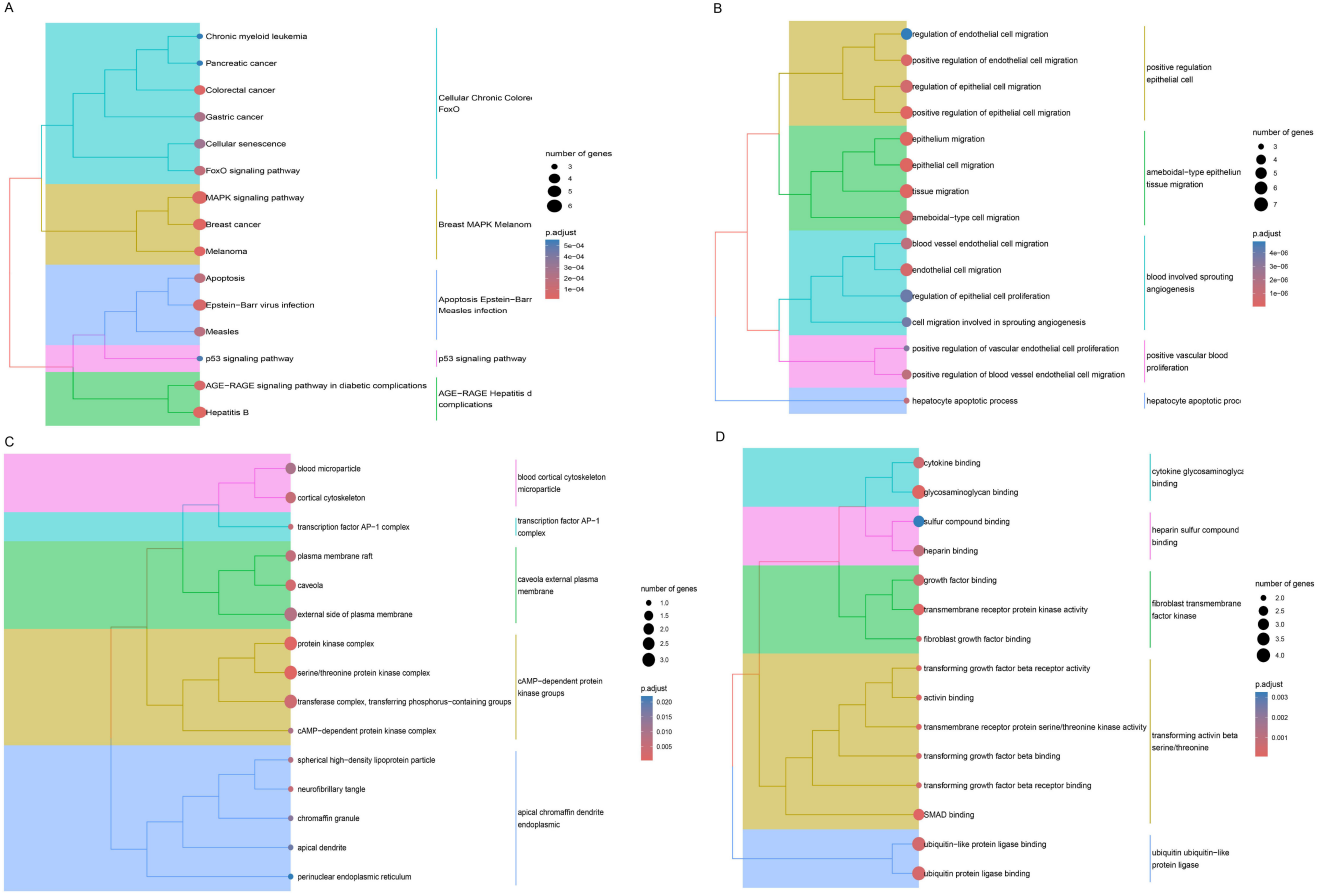
KEGG and GO enrichment analysis. **(A)** Cluster tree diagram of KEGG enrichment analysis. The size of the node represents the number of enriched genes. The larger the node, the more enriched genes. The node color indicates the salience of pathway enrichment, with the more red the color, the more significant. **(B)** Cluster tree diagram of GO (BP) enrichment results. **(C)** Cluster tree diagram of GO (CC) enrichment results. **(D)** Cluster tree diagram of GO (MF) enrichment results.

KEGG enrichment analysis revealed that 149 KEGG pathways were markedly enriched (*P*<0.05). The cluster tree map demonstrated that the significant TOP15 pathway, and 21 crucial genes were mainly participated in the MAPK signaling pathway. and the significant TOP15 pathways were visualized by cluster tree diagram, and it was found that 21 candidate genes were mainly involved in MAPK signaling pathway. Breast cancer, Epstein-Barr virus infection, and Hepatitis B pathways (**Figure 3A**). GO enrichment analysis proved that 21 crucial genes were mainly participated in epigenesis migration (BP), cAMP dependent protein kinase (CC), and ubiquitin protein ligase binding (MF) processes (**Figures 3B-D**). Meanwhile, PPI relationships between candidate genes were obtained through the STRING database, resulting in a total of 27 PPI relationships for 17 genes (**Supplementary Figure 1**).

### 3.4 Screening and validation of prognostic biomarkers

**Figure 4:**
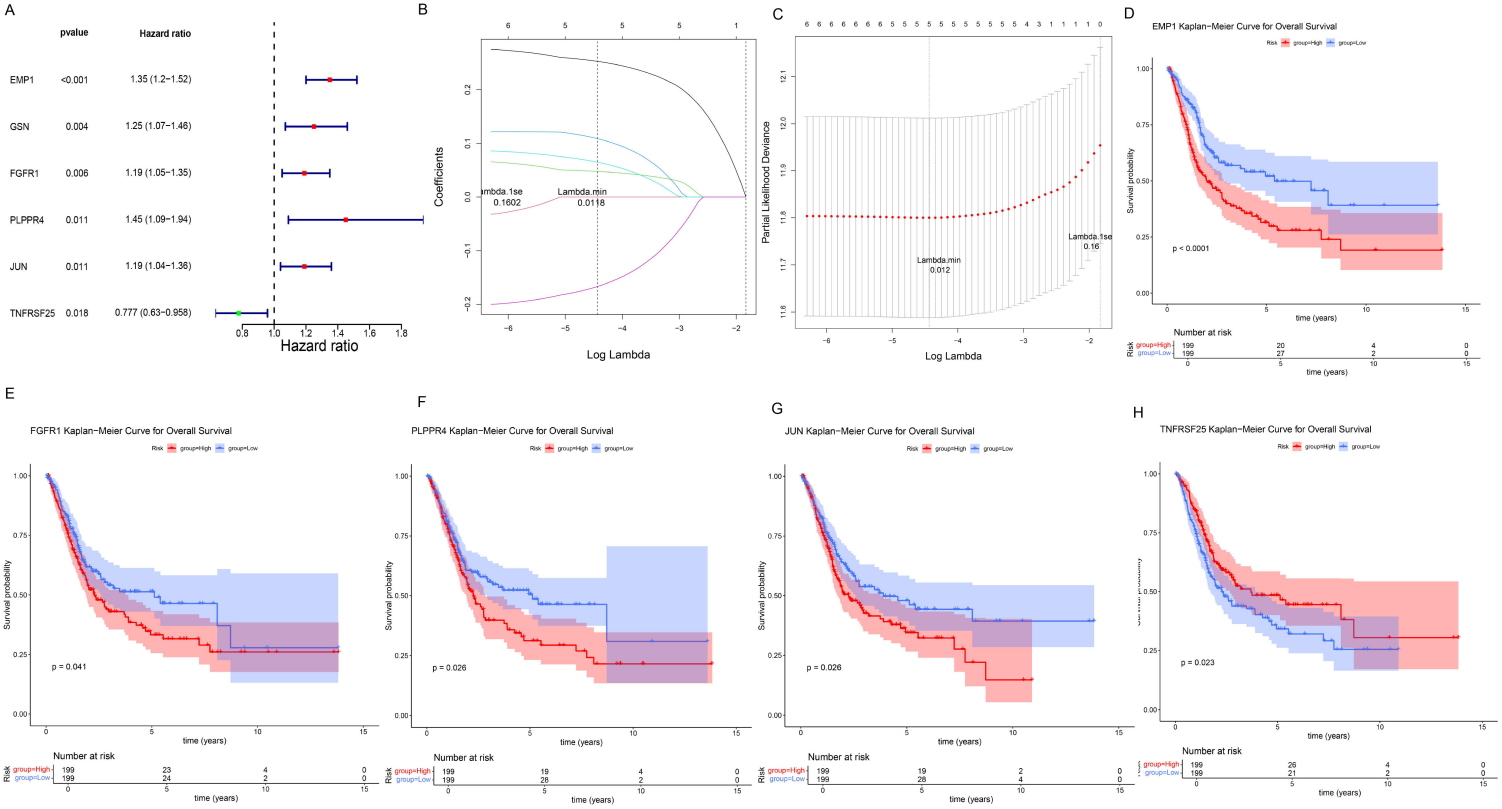
Screening and prognostic value of prognostic biomarkers. **(A)** Forest plot of univariate Cox regression analysis results. The leftmost column displays the selected genes, while the second and third columns present their corresponding P-values and HR (Hazard Ratio) values respectively. The HR values are followed by their 95% confidence intervals in parentheses. In the right-side figure, the red/green squares represent HR values, with the flanking line segments indicating the 95% confidence intervals of the HR values. **(B**, **C)**, LASSO regression analysis results. In Panel **(B)**, the position of the left dashed line indicates the point of minimum cross-validation error. Based on this position (lambda.min), the corresponding log(Lambda) value on the horizontal axis is determined. The top of the panel displays the number of key genes, which is 5. After identifying the optimal log(Lambda) value, the corresponding gene and its coefficient can be located in Panel **(C)**. **(D–H)** K–M survival curves for five prognostic biomarkers, with the horizontal axis representing total survival time (years) and the vertical axis representing survival probability; Red represents the high expression group, blue represents the low expression group. **(D)** EMP1; **(E)** FGFR1; **(F)** PLPPR4; **(G)** JUN; **(H)** TNFRSF25.

Univariate Cox regression analysis of crucial genes in the TCGA-BLCA dataset identified six genes with prognostic significance, including EMP1, GSN, FGFR1, PLPPR4, JUN, and TNFRSF25 (**Figure 4A**). Subsequently, LASSO regression analysis was conducted on six crucial genes, and it was ultimately determined that EMP1, FGFR1, PLPPR4, JUN, and TNFRSF25 genes could serve as prognostic biomarkers (**Figures 4B, C**). In addition, patients were classified into different groups based on the expression of these five prognostic biomarkers, and the analysis revealed survival differences between the different groups (*P*< 0.05, **Figures 4D–H**). Among them, EMP1, FGFR1, PLPPR4 and JUN high expression group had a lower survival rate, while TNFRSF25 high expression group showed the opposite trend (**Figures 4D–H**).

**Figure 5:**
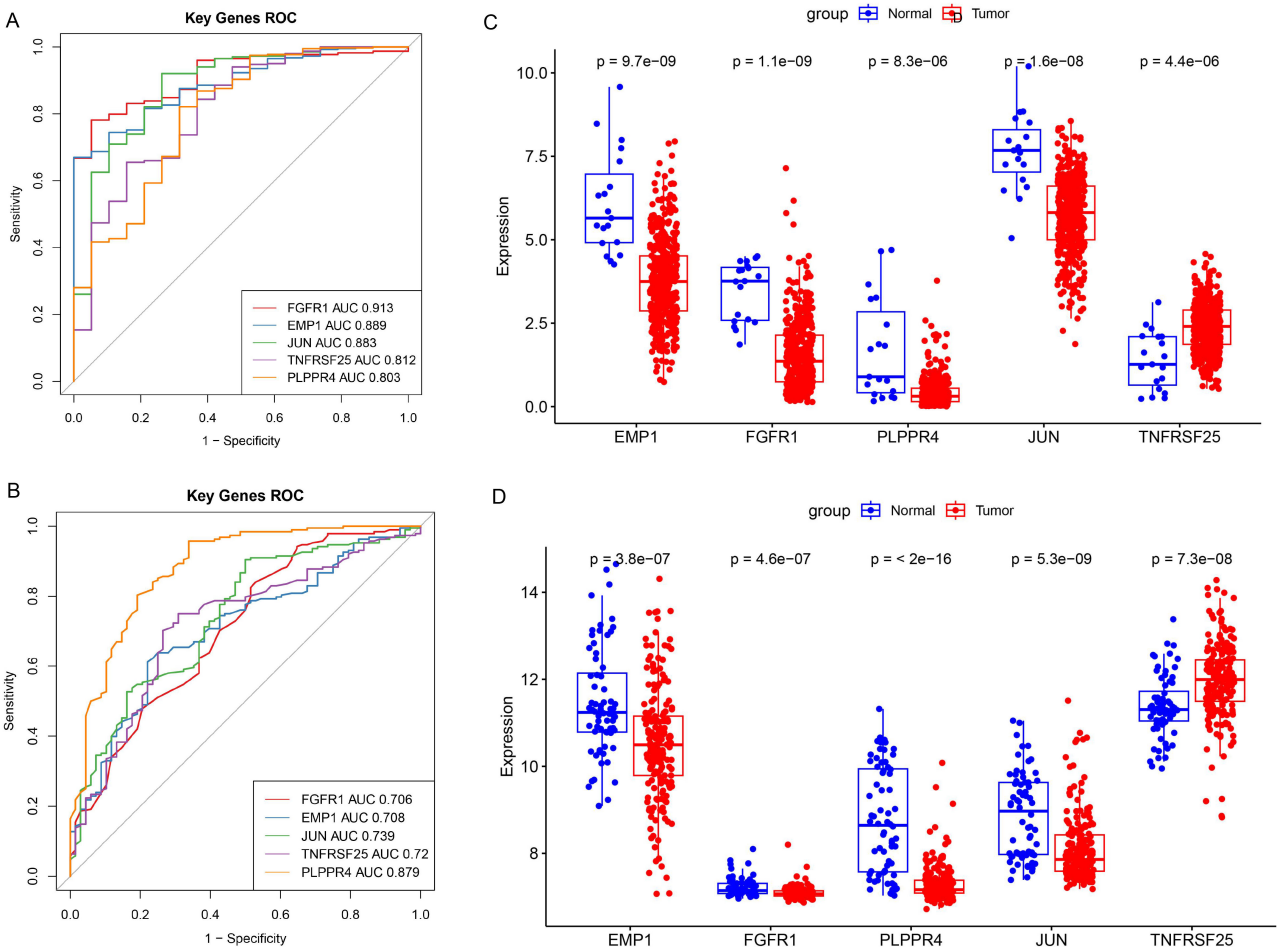
Diagnostic value and expression of prognostic biomarkers. **(A**, **B)**, ROC curve analysis of prognostic biomarkers. The area under the curve is called AUC (Area Under the Curve), which indicates prediction accuracy. The higher the AUC value (i.e., the larger the area under the curve), the higher the prediction accuracy. **(A)** Training set TCGA-BLCA; **(B)** Validation set GSE13507. **(C, D)**, Differential expression analysis of prognostic biomarkers. **(C)** Training set TCGA-BLCA; **(D)** Verification set GSE13507.

Draw ROC curves of biomarkers in the training set TCGA-BLCA and assess the diagnostic significance of each biomarker. The results showed that all five prognostic biomarkers had good diagnostic value (AUC>0.8, **Figure 5A**) in the training set TCGA-BLCA. At the same time, the five prognostic biomarkers also had good diagnostic value in the validation set (AUC>0.7, **Figure 5B**). Additionally, the expressions of EMP1, FGFR1, PLPPR4, JUN, and TNFRSF25 were significantly different between the training set and the validation set, and the difference trend was consistent (*P*<0.05, **Figures 5C, D**).

### 3.5 GSEA and GSVA enrichment analysis

**Figure 6:**
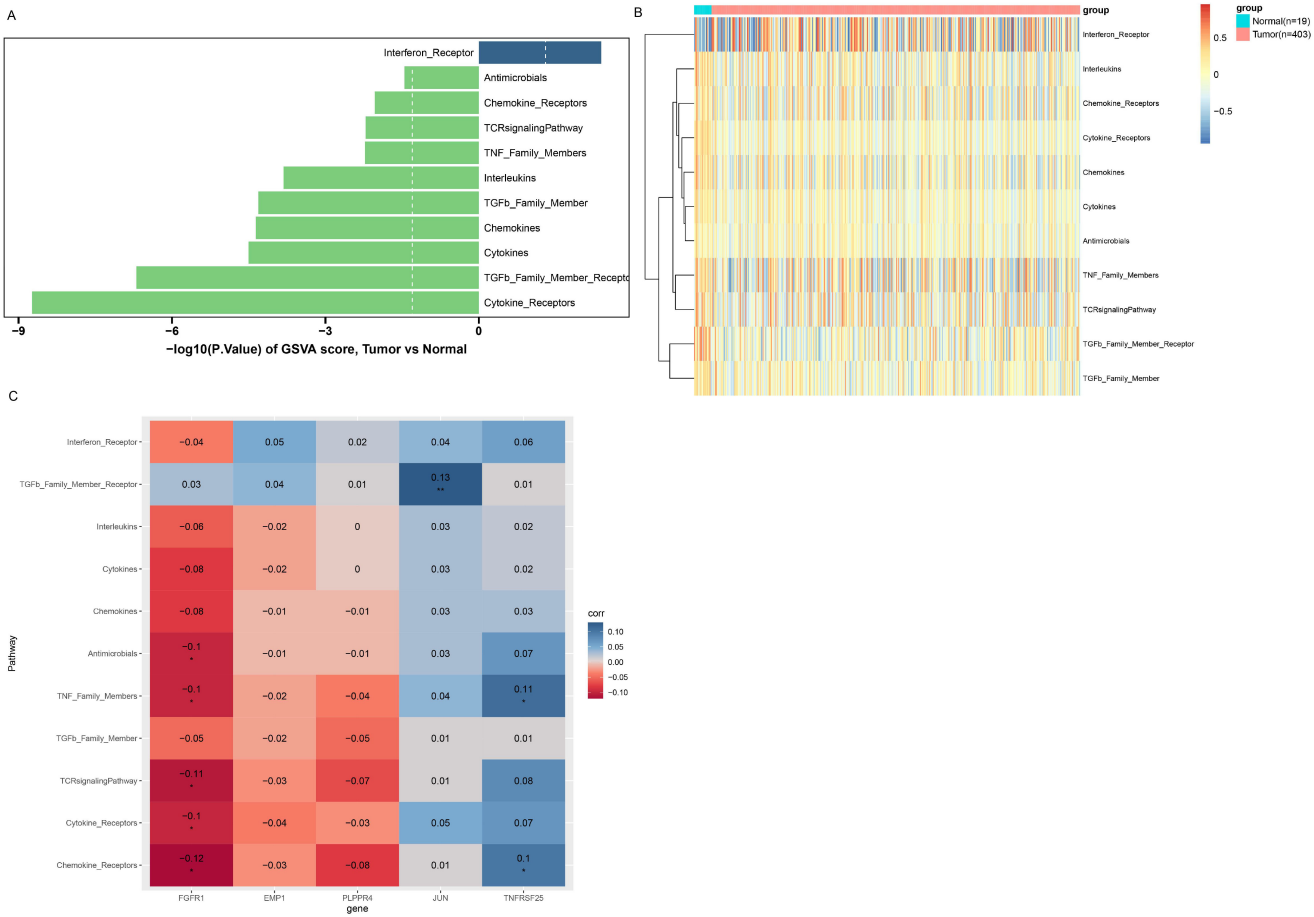
GSVA enrichment analysis. **(A)** Immune response pathways with significant differences between groups. The left column is the immune response pathway downregulated in the Tumor group. The right column is the immune response pathway upregulated in the Tumor group. Longer columns indicate more significant differences in this pathway between groups. **(B)** enrichment scores of immune response pathways with significant differences between groups. **(C)** Correlation between immune response pathways with significant differences and prognostic biomarkers. The horizontal axis represents biomarkers, and the vertical axis represents immune response pathways. Color indicates the magnitude of correlation, red indicates negative correlation, blue indicates positive correlation, the darker the color, the stronger the correlation. *P<0.05; **P<0.01.

The GSEA algorithm was employed to further investigate the potential biological mechanisms of prognostic biomarkers and visualize the TOP10 KEGG pathways (**Supplementary Figures 2A–E**). Among them, FGFR1 mainly participates in ribosome, cytoskeleton in muscle cells, oxidative phosphorylation, cGMP-PKG signaling pathway and other pathways (**Supplementary Figure 2E**). Eleven immune response pathways with significant differences between different groups were screened by GSVA algorithm, among which the Interferon-Receptor pathway was upregulated in the cancer group, while the remaining 10 response pathways were downregulated in the cancer group (**Figure 6A**). Besides, the enrichment scores of 11 immune response pathways are shown in **Figure 6B**. Moreover, correlation analysis between the five genes and differential immune response pathways revealed that FGFR1 was mainly negatively correlated with Cytokine Receptors, TCR signaling Pathway, Antimicrobials and other pathways (**Figure 6C**).

### 3.6 Immune microenvironment analysis

**Figure 7:**
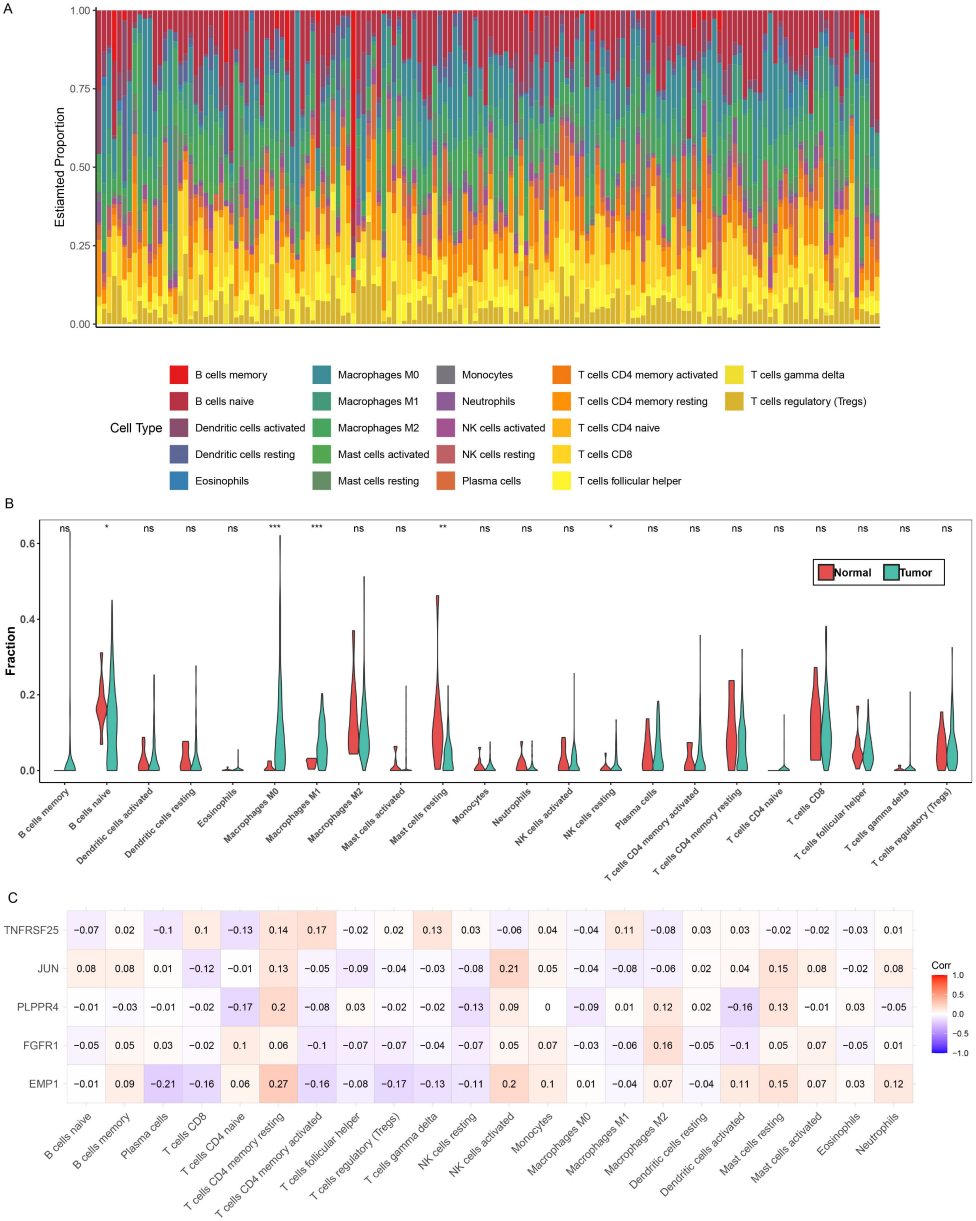
Immune microenvironment analysis. **(A)** Relative proportions of 22 immune cells in different samples. **(B)** Differences in the relative proportions of the 22 immune cells in the different groups, *P<0.05; **P<0.01; ***P<0.001. **(C)** Correlation between 22 immune cells and biomarkers. Red indicates positive correlation, blue indicates negative correlation, the darker the color, the stronger the correlation.

First, **Figure 7A** presents the relative proportions of the 22 immune cells in each tumor sample. Next, an analysis of the relative proportions of 22 immune cells found that the five immune cells, Mast cells resting, Macrophages M1, Macrophages M0, B cells naive and NK cells resting, showed significant differences between different groups (**Figure 7B**). Subsequently, correlation analysis between the five biomarkers and 22 types of immune cells revealed obvious positive relationship between EMP1 and CD4 memory resting of T cells, and obvious negative relationship with plasma cells (**Figure 7C**).

### 3.7 Validation of prognostic biomarker expression

**Figure 8:**
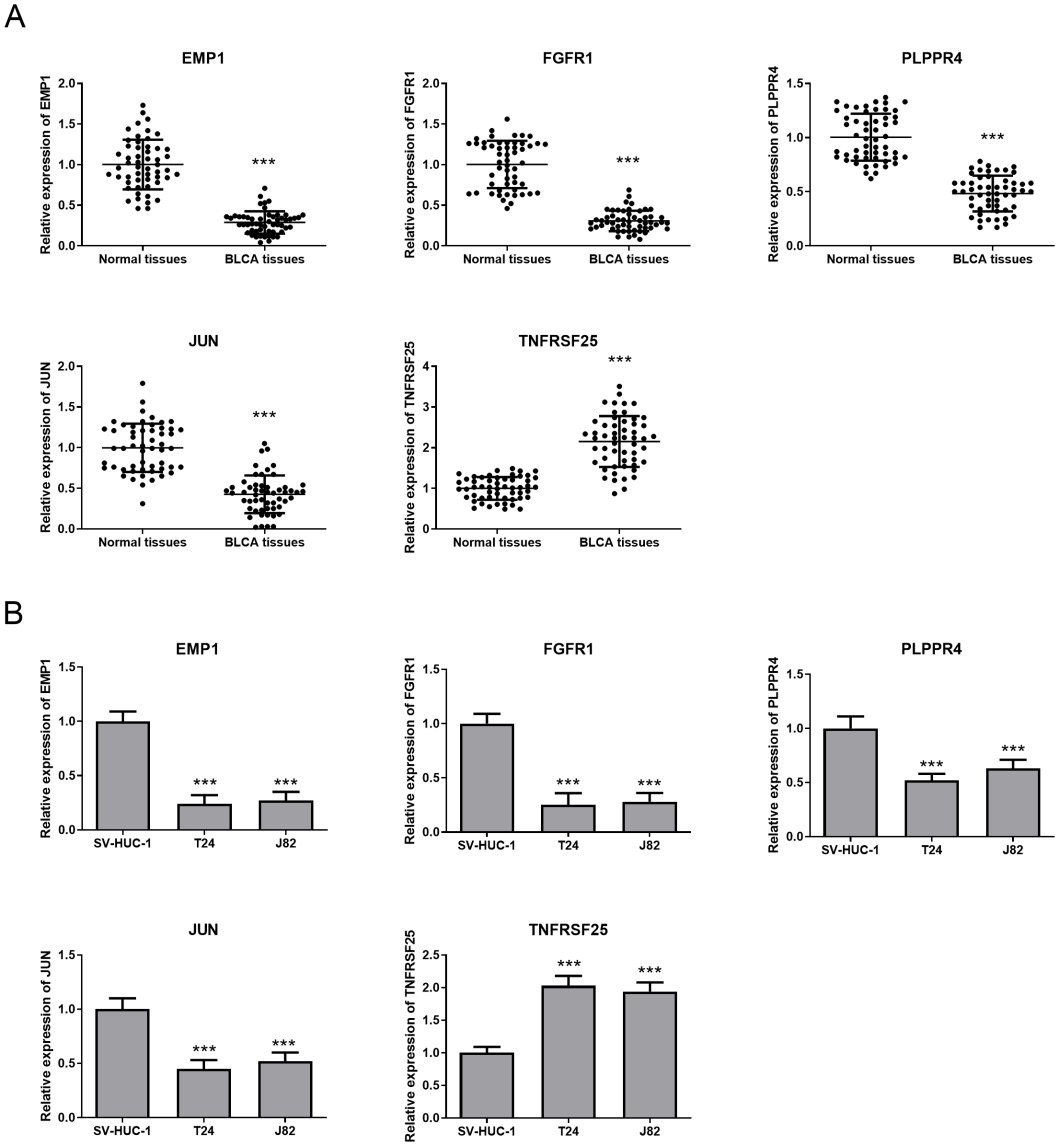
Expression of prognostic biomarkers in BLCA tissues and cells. **(A)** QRT-PCR was used to detect the expression of five prognostic biomarkers in BLCA tissues and adjacent tissues. **(B)** The expression of five prognostic biomarkers in SV-HUC-1,T24 and J82 cell lines was detected by qRT-PCR. ***P<0.001.

Here, we examined the expression of EMP1, FGFR1, PLPPR4, JUN, and TNFRSF25 in BLCA tissue specimens and two BLCA cell lines, T24 and J82. The results pointed out that EMP1, FGFR1, PLPPR4, and JUN were all markedly decreased in BLCA tissue specimens and cell lines (T24 and J82) compared with adjacent normal tissue specimens and SV-HUC-1 cells (**Figures 8A, B**), while TNFRSF25 showed the opposite trend (**Figures 8A, B**). This is consistent with the expression trend of bioinformatics analysis. The expression of five biomarkers was more significant in T24 cells, and based on this difference, T24 cells were selected for subsequent research. Previous studies have shown that the FGFR family plays a key role in the occurrence and development of BLCA (26, 27), but most of the existing literature focuses on the carcinogenic effect of FGFR3, while the prognostic value of FGFR1 in BLCA remains unknown. In addition, verification experiments showed that the expression of FGFR1 in BLCA tissues and cell lines changed most significantly compared with other biomarkers. Therefore, FGFR1 was selected for subsequent research.

### 3.8 FGFR1 overexpression inhibited T24 cell proliferation, migration and invasion

**Figure 9:**
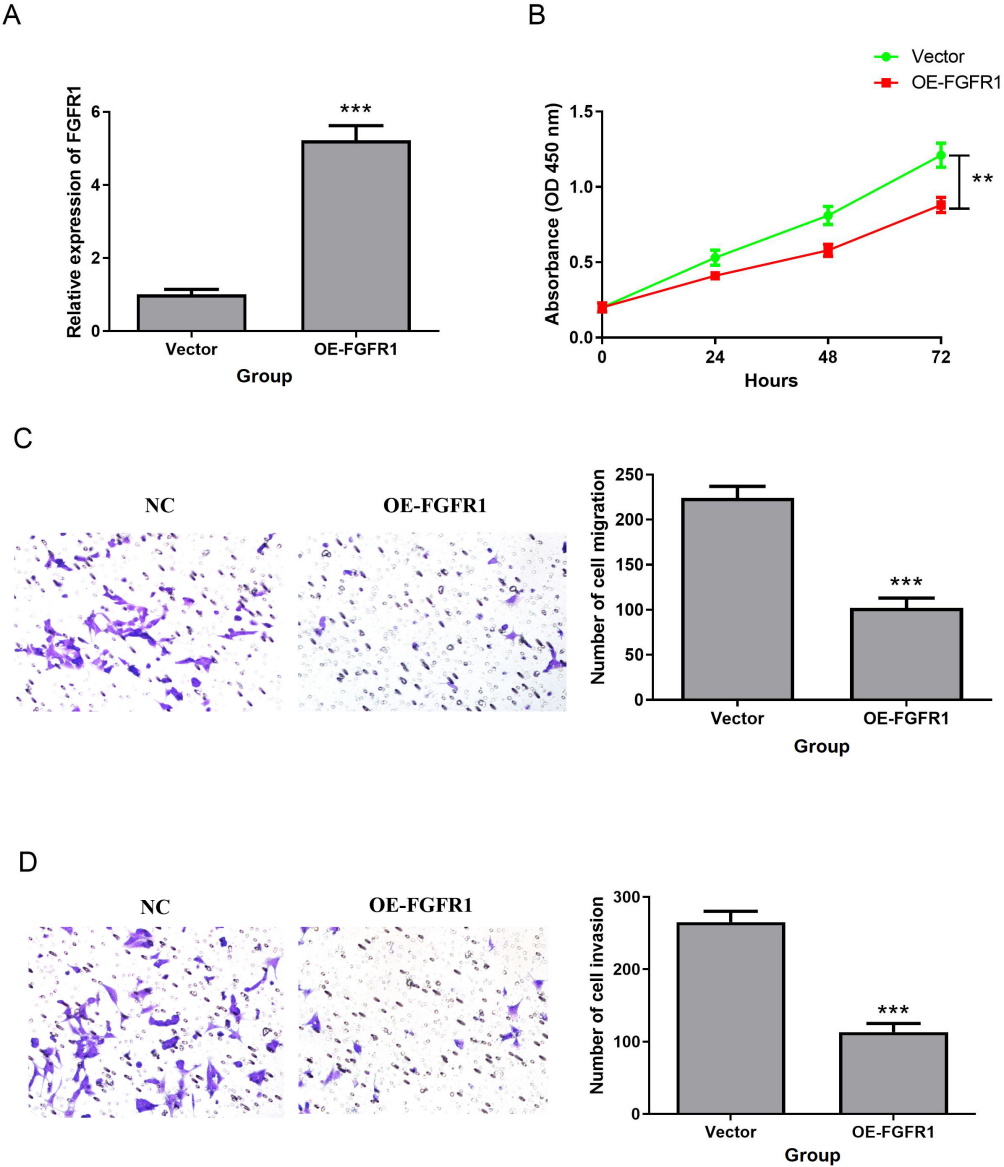
FGFR1 overexpression inhibited T24 cell proliferation, migration and invasion. **(A)** QRT-PCR was used to detect the expression of FGFR1 in T24 cell line transfected with OE-FGFR1. **(B)** Effect of FGFR1 overexpression on T24 cell proliferation. **(C**, **D)**, Transwell to verify the effect of upregulated FGFR1 expression on the migration **(C)** and invasion **(D)** abilities of T24 cells. **P<0.01; ***P<0.001.

We investigated the effect of FGFR1 on the biological function of T24 cells. OE-FGFR1 and its NC were transfected into T24 cells, and qRT-PCR confirmed the successful transfection, and the transfection NC did not change the expression level of FGFR1 (**Figure 9A**). Besides, overexpression of FGFR1 markedly reduced the proliferation, migration, and invasion abilities of T24 cells (**Figures 9B–D**). All data suggested that upregulation of FGFR1 expression inhibits T24 cell biological processes.

### 3.9 FGFR1 inhibits the biological function of T24 cells by activating the cGMP PKG pathway

**Figure 10:**
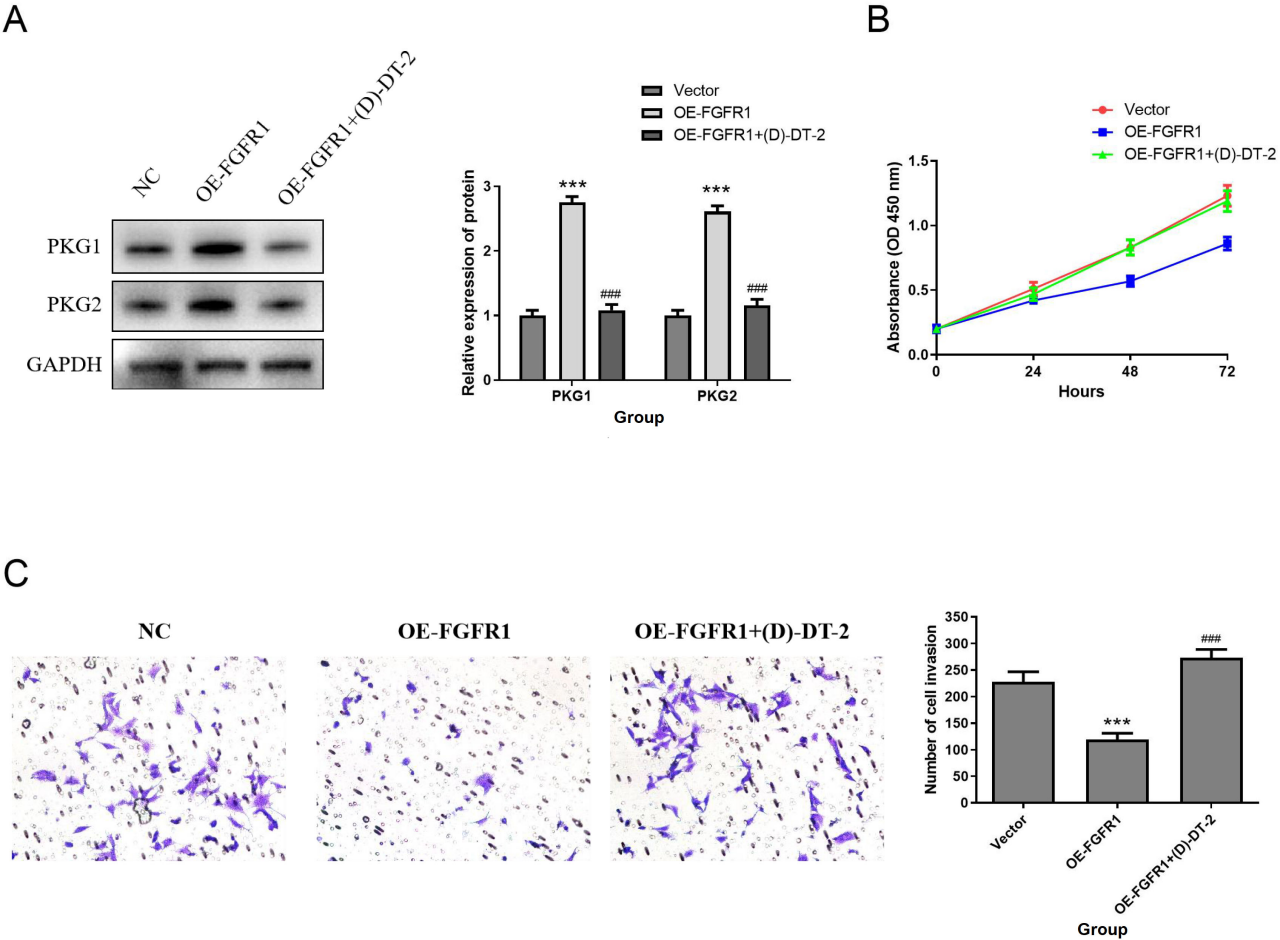
FGFR1 inhibits the proliferation, migration and invasion of T24 cells by activating the cGMP-PKG pathway. **(A)** PKG1 and PKG2 protein expression was determined by Western Blotting. **(B–D)** Effect of PKG inhibitor **(D)**-DT-2 on T24 cell proliferation **(B)**, migration **(C)**, and invasion **(D)**. Compared with NC group, ***P<0.001; Compared with OE-FGFR1 group, ^###^ *P*<0.001.

**Supplementary Figure 2E** showed a clear relationship between FGFR1 and cGMP-PKG signaling pathway. Therefore, western blotting was applied to detect the effect of FGFR1 on the cGMP/PKG pathway. The data indicated that the protein levels of PKG1 and PKG2 were obviously upregulated in the FGFR1 overexpression group. However, PKG inhibitor (D)-DT-2 inhibited this increase (D)-DT-2 (**Figure 10A**). Subsequently, we evaluated the impact of PKG inhibitor (D)-DT-2 on the biological functions of T24 cells. The data revealed that overexpression of FGFR1 reduced cell proliferation (**Figure 10B**) and invasion (**Figure 10C**) compared to the NC group, but this inhibition was restored after the addition of PKG inhibitor (D)-DT-2 (**Figures 10B, C**). Therefore, we hypothesized that FGFR1 may inhibit the cellular biological processes of T24 cells by activating the cGMP-PKG pathway.

## 4 Discussion

BLCA is a malignant cancer with high mortality. At present, there are a variety of treatment methods, the treatment effect is not ideal due to tumor recurrence, metastasis, chemotherapy drug resistance and other reasons, and the clinical outcome of patients is very unsatisfactory (28, 29). Accordingly, the development and identification of effective prognostic biomarkers are very helpful to optimize treatment and improve the prognosis of BLCA patients. BLCA is the first cancer for which PDT has been approved for clinical treatment. PDT not only directly kills tumor cells, but also induces immunogenic cell death (ICD), improving the efficacy of anti-tumor immunotherapy (30, 31). According to reports, many ARGs are associated with the occurrence and prognosis of BLCA (32). However, the prognostic significance of ARGs-PDTRGs in BLCA remain unclear. For this study, based on the BLCA data in TCGA, 21 candidate genes of ARGs-PDTRGs were screened by a variety of bioinformatics methods. Five candidate genes interrelated to the prognosis of BLCA were further identified, and the prognostic value and mechanism of these five candidate genes in BLCA were systematically analyzed.

MAPK signaling plays a crucial role in tumor development and immunotherapy, and prognostic genes related to the MAPK pathway can predict the prognosis of BLCA patients (33). MAPK signaling marks two different subpopulations of tumor cells in BLCA, and inhibiting MAPK signaling can suppress tumor growth (34). Moreover, a meta-analysis showed that Epstein-Barr virus (EBV) is significantly associated with the occurrence of BLCA (35). The current study revealed that 21 candidate genes were mainly involved in MAPK signaling pathway, Breast cancer, Epstein-Barr virus infection and Hepatitis B pathways. So that, we speculate that these genes may participate in the occurrence and development of BLCA via the above pathways, but this conclusion still needs further verification.

For this research, five ARGs-PDTRGs (EMP1, FGFR1, PLPPR4, JUN, TNFRSF25) were finally identified as BLCA prognostic biomarkers after a series of bioinformatics analyses. K-M survival curve revealed obvious survival differences between groups. ROC curve analysis showed that these five biomarkers had high diagnostic value. Song et al. (36) established an ARGs model, which can predict the clinical outcomes and immunotherapy of BLCA. Another study (37) constructed a BLCA prognostic signature based on 17 ARGs, which could predict the clinical outcomes of BLCA cases and lay the foundation for individualized treatment of cases. A total of 5 prognostic markers were screened in current research, including EMP1, FGFR1, PLPPR4, JUN and TNFRSF25. Among them, EMP1 is a key gene indicating the M1/M2 ratio, and its upregulation suggests a short survival of BLCA patients. Besides, epithelial membrane protein 1 (EMP1) expression level can be used as an indicator of BLCA cell proliferation, metastasis and immunotherapy efficacy (38). Fibroblast growth factor receptor 1 (FGFR1), a member of the FGFR family, is associated with the proliferation of BLCA cells (39). Phospholipid phosphatase related 4 (PLPPR4) is a member of the lipid phosphophosphatases superfamily, which can regulate neural development by affecting neuronal plasticity through mTOR signaling pathway (40). Feng et al. (41) screened nine dephosphorylation related genes, including PLPPR4, and constructed a prognostic signature related to papillary renal cell carcinoma, which could accurately predict the survival outcomes of PRCC cases. JUN is a major component of activator protein-1 (42). Han et al. demonstrated that c-Jun is a new bone metastasis marker for luminal type breast cancer and that inhibition of c-Jun effectively inhibited the malignant progression in MCF7-BM cells (43). Tumor necrosis factor receptor superfamily 25 (TNFRSF25) is a T cell co stimulatory receptor and a potential target for cancer therapy. TNFRSF25 agonists can stimulate CD8+T cells and exert anti-tumor effects (44). Overall, all five prognostic markers are potential therapeutic targets for BLCA.

Many researches have found that FGFR1 plays a key role in multiple cancers in humans, including breast cancer and colorectal cancer (45–47). Validation experiments in this study proved that the levels of EMP1, FGFR1, PLPPR4 and JUN were obviously decreased in BLCA tissues and cell lines (T24 and J82), while TNFRSF25 showed an opposite trend, compared with those in para-cancerous normal tissues and normal urothelial SV-HUC-1 cells. Notably, previous studies have shown that the FGFR family plays a key role in the occurrence and development of BLCA (26, 27), but most of the existing literature focuses on the carcinogenic effect of FGFR3, while the prognostic value of FGFR1 in BLCA remains unknown. In addition, verification experiments showed that the expression of FGFR1 in BLCA tissues and cell lines changed most significantly compared with other biomarkers. Therefore, FGFR1 was selected for subsequent research. This research revealed that FGFR1 was mainly participated in ribosome, cytoskeleton in muscle cells, oxidative phosphorylation, cGMP-PKG signaling pathway and other pathways. This finding significantly expands the functional spectrum of FGFR1, as existing literature primarily documents the role of the cGMP-PKG pathway in prostate cancer, ovarian cancer, and other malignancies (48, 49), while its regulatory mechanisms in BLCA remain an emerging field of research. A previous study revealed that metformin inhibits the progression of castration-resistant prostate cancer by modulating PDE6D-induced purine metabolic alterations and activating the cGMP/PKG pathway (48). Another study demonstrated that PTTG1 promotes M2 macrophage polarization through the cGMP/PKG signaling pathway and facilitates epithelial-mesenchymal transition (EMT) progression in human epithelial ovarian cancer cells (49). Through systematic *in vitro* experiments and signaling pathway analysis, we have demonstrated for the first time that FGFR1 significantly inhibits the biological functions of T24 cells by specifically activating the cGMP-PKG pathway. These groundbreaking findings not only indicate the involvement of FGFR1 in the pathogenesis of BLCA, but more importantly, reveal a distinct signaling network regulated by this pathway in BLCA. In summary, this study is the first to elucidate the critical regulatory role of FGFR1 in the initiation and progression of BLCA through the cGMP-PKG signaling pathway, thereby identifying a novel potential therapeutic target for BLCA treatment.

In the past decade, new therapies such as immunotherapy have driven the progress of BLCA treatment. Immunotherapy can enhance its ability to clear cancer cells and strengthen the body’s anti-tumor immune response. Some immunotherapy drugs, such as PD-L1, PD-1 and CTLA-4, have been applied in clinical practice (50, 51). At present, the cell types, pathways and processes involved in anti-tumor immunity are becoming increasingly clear (52). For example, cytotoxic CD4+ T cells can kill autologous tumors in an MHC class II-dependent manner and are inhibited via Tregs (53). In this study, GSVA enrichment analysis of five prognostic biomarkers was performed, and the Interferon Receptor response pathway was found to be up-regulated in the tumor group, while the remaining 10 response pathways were down-regulated in the tumor group. Further analysis showed that FGFR1 was negatively correlated with Cytokine Receptors, TCR signaling Pathway, Antimicrobials and other pathways. Tumor cells, immune cells, cytokines, etc. together constitute the tumor microenvironment (TME), among which tumor-related immune cells can be classified into two categories: anti-tumor and pro-tumor (54). B cells are the main effector cells of humoral immunity in TME and play a key role in regulating anti-tumor immune responses (55). Macrophages are a double-edged sword that play a dual role in cancer, both promoting tumorigenesis and killing tumor cells to enhance anti-tumor response (56). NK cells have cytotoxic functions similar to CD8+ T cells and are the first line of natural defense, allowing to kill some tumor and virus-infected cells (57, 58). In this study, we found that Mast cells resting, Macrophages M1, Macrophages M0, B cells naive and NK cells resting have significant differences among different groups. This suggests that these immune pathways and immune cells may be participated in the occurrence and development of BLCA, and provide a reliable target for immunotherapy of BLCA patients.

PDT, as a known physical therapy capable of inducing apoptosis in tumor cells, has a natural correlation between its target sites and tumor prognosis genes (59, 60). Therefore, the initial gene screening in this study focused on PDT and apoptosis, ultimately identifying five prognostic biomarkers (EMP1, FGFR1, PLPPR4, JUN, TNFRSF25). All five genes are PDTRGs and ARGs. Although this study did not directly conduct PDT experiments, the screening of FGFR1 derived from the PDT-related gene network, and the discovery of the cGMP-PKG pathway, provides a new perspective regarding the molecular mechanism of PDT in treating BLCA. A previous study found that in the SW837 colorectal cancer cell model, PDT treatment using aminolevulinic acid (ALA) as a photosensitizer was significantly associated with alterations in the cGMP-PKG signaling pathway (61). PDT induces the production of a large amount of reactive oxygen species (ROS) in tumor cells through laser irradiation of a photosensitizer. The resulting cytotoxicity selectively targets tumor cells, inducing apoptosis (62). Moreover, the generation of ROS is significantly associated with the cGMP/PKG signaling pathway (63). This suggests that cGMP-PKG may be one of the conserved pathways of PDT action. This study establishes FGFR1/cGMP-PKG as a novel regulatory axis in BLCA, providing a fundamental basis for subsequent PDT combination strategies. The decision to defer PDT experiments at this stage represents a rigorous scientific approach. Initial validation of the independent role of this novel pathway avoids confounding variables. Subsequent investigations will involve establishing FGFR1-overexpressing cell models subjected to PDT treatment and conducting orthotopic murine model-based photodynamic-drug combination experiments to further validate this mechanism.

Although our study has achieved some satisfactory results, there are still some limitations that cannot be ignored. First, we collected a small number of clinical samples, which may cause some impact on the accuracy of the results. Secondly, we have only preliminarily explored the molecular mechanism of one prognostic marker in BLCA, and the other genes need to be further studied. In addition, follow-up and prognostic analysis of recruited patients were not performed due to time constraints. Therefore, we will expand the sample size and collect clinical data of patients to further investigate the prognostic significance and molecular mechanism of these five prognostic biomarkers in BLCA, in order to provide effective personalized treatment or targeted therapy for patients.

## 5 Conclusion

In conclusion, this study successfully screened five important ARGs-PDTRGs (EMP1, FGFR1, PLPPR4, JUN, TNFRSF25) as BLCA prognostic biomarkers and analyzed their roles in BLCA by multiple bioinformatics methods. Experimental studies have confirmed that FGFR1 inhibits the proliferation, migration and invasion of T24 cells via activating the cGMP-PKG pathway, which provides a novel potential target for clinical diagnosis, treatment and prognosis of BLCA.

## Data Availability

The raw data supporting the conclusions of this article will be made available by the authors, without undue reservation.
