# Supplementary material for: Prognostic value and molecular mechanism of photodynamic therapy and apoptosis related gene FGFR1 in bladder cancer
Source: Front Oncol. 2025 Sep 10;15:1578695. doi: 10.3389/fonc.2025.1578695 (PMC12457120; doi:10.3389/fonc.2025.1578695)
Supplement: Supplementary file 1 [file DataSheet1.docx]

***Supplementary Material***


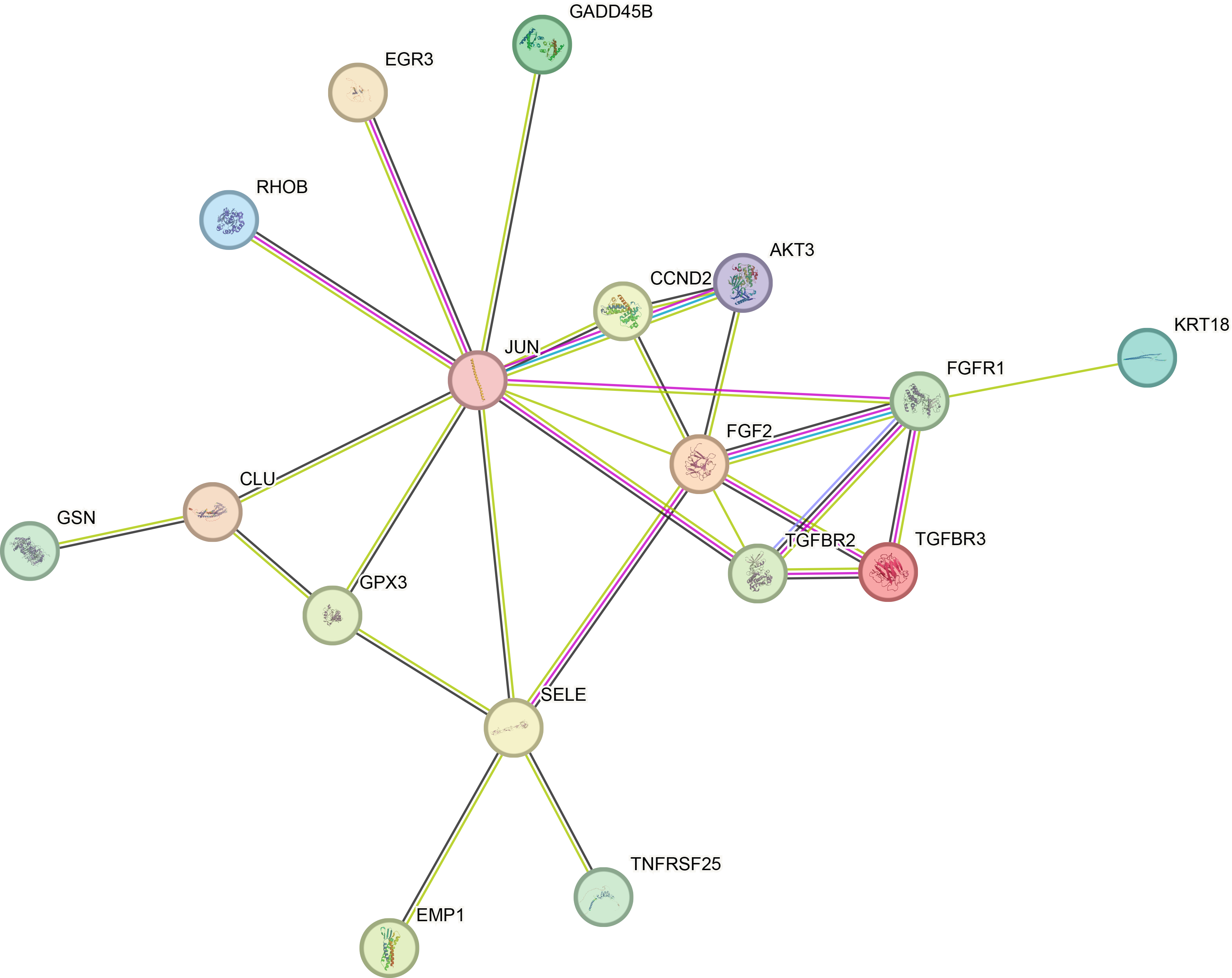


**Supplementary Figure 1.** PPI network map of candidate genes.


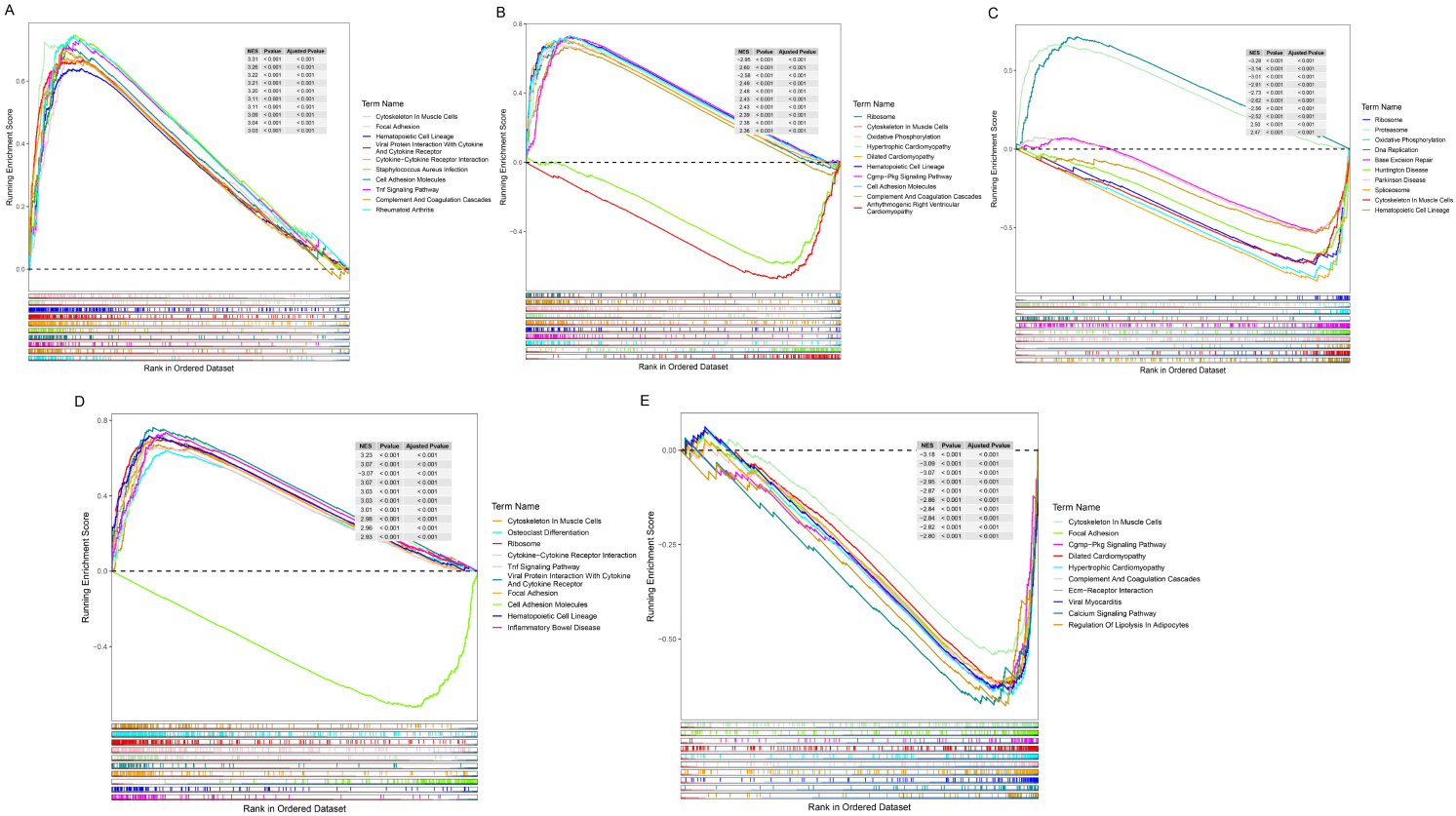


**Supplementary Figure 2.** GSEA analysis of prognostic biomarkers. A, EMP1; B, FGFR1; C, PLPPR4; D, JUN; E, TNFRSF25.
